# The cGAS/STING Pathway: A Novel Target for Cancer Therapy

**DOI:** 10.3389/fimmu.2021.795401

**Published:** 2022-01-03

**Authors:** Yu Gan, Xiaoying Li, Shuangze Han, Qi Liang, Xiaoqian Ma, Pengfei Rong, Wei Wang, Wei Li

**Affiliations:** ^1^ Department of Radiology, The Third Xiangya Hospital of Central South University, Changsha, China; ^2^ Cell Transplantation and Gene Therapy Institute, The Third Xiangya Hospital, Central South University, Changsha, China

**Keywords:** cGAS-STING, cancer, immunotherapy, STING agonists, combined therapy

## Abstract

As a DNA receptor, cyclic GMP-AMP synthase (cGAS) plays a crucial role in the immune system by recognizing abnormal DNA in the cytoplasm and activating the stimulator of interferon genes (STING) signaling pathway. This signaling cascade reaction leads to an immune response produced by type I interferon and other immune mediators. Recent advances in research have enhanced our current understanding of the potential role of the cGAS/STING pathway in anticancer therapy; however, in some cases, chronic STING activation may promote tumorigenesis. The present review article discusses the biological mechanisms of the cGAS/STING pathway, its dichotomous role in tumors, and the latest advances with respect to STING agonists and antagonists.

## 1 Introduction

Cancer is the leading cause of mortality globally and a prominent obstacle in prolonging human life expectancy. Classical therapies, such as surgical resection, radiotherapy and chemotherapy, remain the most common treatment regimens for cancer. However, not all cancers respond to classical treatments, which has prompted further research to discover novel treatment strategies (1). Over the past few decades, our understanding of immunology has increased, providing hope for success in cancer immunotherapy (2). Cyclic GMP-AMP (cGAMP) synthase (cGAS) is a cytoplasmic DNA sensor that can activate the stimulator of interferon (IFN) genes (STING) protein to subsequently induce a protective immune defense against various DNA-containing pathogens and provides antitumor immunity (3). The present review article aimed to summarize the immune response mediated by cGAS and discuss its dichotomous role in tumor development and the application of STING in antitumor therapy.

## 2 Overview of the cGAS/STING Pathway

### 2.1 Structure and Signal Transduction of the cGAS/STING Pathway

cGAS is an innate immune sensor that can recognize various cytoplasmic double-stranded (ds)DNAs, including viral, bacteria, mitochondrial, micronuclei and retroelement origin DNA. The C-terminal region of cGAS exhibits nucleotide transferase activity (4), binding to dsDNA can lead to profound conformational alterations in cGAS (5), primarily affecting catalytic pockets. Subsequently, ATP and GTP in this pocket can be used as substrates to synthesize GAMP (6). Another important N-terminal domain in cGAS is responsible for maintaining the liquid phase of dsDNA and cGAS. The non-specific ion interaction between the positively charged N-terminal of cGAS and the structural contact between the C site and dsDNA enforce cGAS/DNA liquid-liquid phase separation (LLPS) (7), which is beneficial for dimerization and protects DNA from the degradation of three-prime repair exonuclease 1 (TREX1) to promote cGAS activity (8).

**Figure 1:**
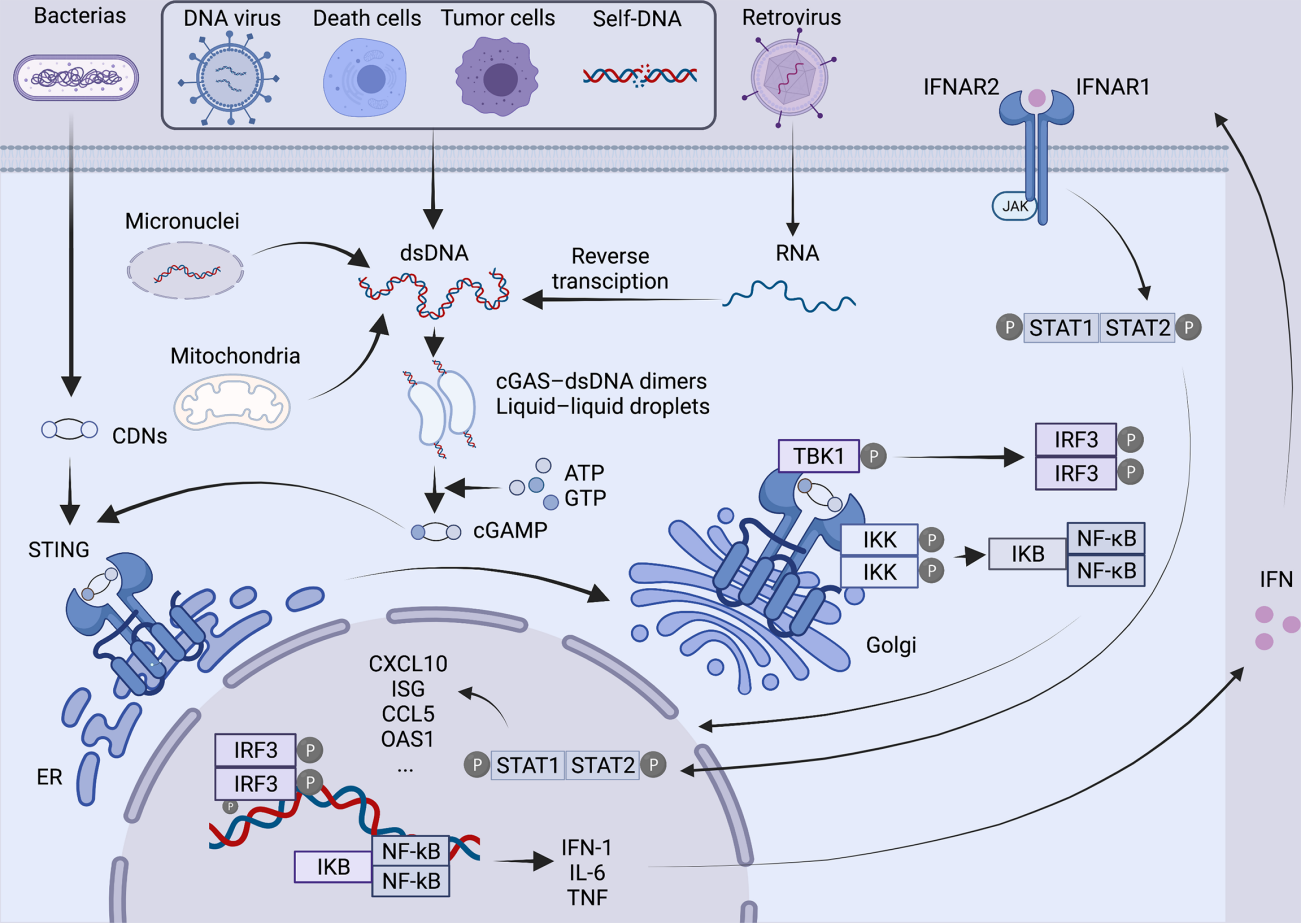
Overview of the cGAS-STING pathway. In the presence of various cytoplasmic DNAs, as a natural immunosensor, cGAS can bind to dsDNA to form a 2:2 cGAS-dsDNA complex, generating a second messenger cGAMP, which activates the STING protein at the endoplasmic reticulum. STING is then transferred from the endoplasmic reticulum *via* ERGIC to the Golgi apparatus, palmitoylated at two cysteine residues Cys88 and Cys91. Modified STING recruits TBK1 and IRF3. At the same time, STING also binds and stimulates IKK, triggering the transcriptional activation of NF-kB. Ultimately, it regulates the expression and secretion of pro-inflammatory cytokines such as IFN. Binding of IFN to IFNAR1/IFNAR2 activates JAK1, phosphorylates STAT, and induces the expression of ISG. Created with BioRender.com.

As a second messenger, cGAMP is detected by the cyclic-dinucleotide sensor, STING (4), a ~40-kDa dimer transmembrane protein (9) in the endoplasmic reticulum (ER). Then STING is transported to the Golgi apparatus through the ER-Golgi intermediate compartment and initiates the downstream signaling cascade (10). After reaching the Golgi apparatus, STING is palmitoylated at two cysteine residues (Cys88 and Cys91) (11), recruiting TANK-binding kinase 1 (TBK1), which in turn phosphorylates the C-terminal of STING and recruits IFN regulatory factor 3 (IRF3). Moreover, STING can bind and stimulate IκB kinase to trigger the transcriptional activation of NF-κB (9), which eventually regulates the expression and secretion of pro-inflammatory cytokines, including IFN-α and IFN-β (12). Subsequently, IFN binding with heterodimeric IFN receptor IFNAR1/IFNAR2 activates Janus kinase 1 (JAK1), which can phosphorylate members of the signal transducer and activator of transcription (STAT) family and induces the expression of IFN-stimulated genes (ISGs) (**Figure 1**). A number of ISGs control viral, bacterial and parasite infections by directly targeting pathogen life cycle pathways and functions (13) .

### 2.2 Activation of the cGAS/STING Signaling Pathway

Pathogen infection, self-DNA damage and tumor DNA are three critical factors that induce cGAS/STING signal activation. The majority of DNA viruses, such as human cytomegalovirus (HCMV), Herpes simplex virus type 1 (HSV-1) (14) and hepatitis B virus, can activate the cGAS/STING pathway and initiate an antiviral immune response. The Dengue RNA virus has also been shown to induce cytoplasmic exposure of mitochondrial DNA (mtDNA) in infected cells to induce cGAS/STING signals (15). It has been shown that under conditions of chronic STING activation, high ISG expression in cancer cells may impose a transcriptional state on the tumor that is used to respond to aberrant dsRNA accumulation due to increased sensor levels (MDA5, RIG-I, and PKR), suggesting that cGAS may act as an indirect sensor of dsRNA and exert anti-tumor effects (16).

DNA is replicated with immense precision during each cell division cycle in normal cells, and genome maintenance systems will monitor and resolve DNA defects in real-time. The DNA damage response (DDR) can counteract endogenous and exogenous injuries and maintain genomic integrity by activating a complex signaling network that promotes transient cell cycle arrest and DNA repair. When DNA damage exceeds the ability of repair, DDR can trigger cell senescence or apoptosis (17). Micronuclei, small nuclear-like bodies composed of chromosome fragments wrapped in fragile nuclear membranes, are traditional biomarkers of DNA damage and chromosome instability (18). Due to the lack of a stable nuclear membrane, the micronucleus envelope can easily break and expose its genome contents to the cytoplasm (19), triggering the cGAS/cGAMP/STING pathway (20). However, during normal mitosis, when the nuclear membrane ruptures and the chromosome DNA expose to cytoplasmic, it is difficult for the chromosome to recognize cGAS. Existing evidence suggests that this is due to the N-terminal hyperphosphorylation and the inhibition of oligomerization caused by chromatin tethering (21).

Cancer cells have common characteristics. In addition to the classic 10 characteristics (22), a group of other markers has been proposed to describe the stress phenotype of cancer cells, including metabolic stress, mitotic stress, oxidative stress and DNA damage stress (23). Under these extreme stress conditions, nuclear and mtDNA are very fragile and are easily destroyed. At the sites of stalled replication forks, DNA structure-specific endonuclease MUS81 can cleave aberrant DNA structures, leading to the accumulation of DNA in the cytoplasm (24). In addition, the chromosome missegregation in cancer cells ultimately leads to the leakage of DNA in the form of micronuclei, chromatin fragments and/or free telomere DNA, triggering the cGAS/STING signaling pathway (25). Exposure to ionizing radiation (IR) or treatment with chemotherapeutic drugs, such as platinum drugs, can also induce DNA double-strand breaks (DSBs) according to a similar mechanism and can subsequently activate the cGAS/STING signaling pathway. In addition to nuclear DNA leakage, malignant tumor cells that have experienced oxidative stress and mitochondrial dysfunction can also release mtDNA into the cytoplasm (26). Under the inhibition of caspase, mtDNA can activate the cGAS/STING signaling by releasing it into the cytoplasm through BAX/BAK-dependent mitochondrial outer membrane permeabilization (27). Furthermore, tumors also acquire mtDNA from the extracellular environment to participate in the cGAS dsDNA-sensing cascade. Cancer cells without mtDNA exhibit a more delayed tumor growth compared with those with mtDNA. Thus, the transfer of mtDNA from host cells in the tumor microenvironment to tumor cells with impaired respiratory function can help reconstruct respiration and initiate the formation of the tumor (28). Genomic substrates from other sources, such as apoptosis-derived DNA, exosomes and transposons, may also induce cGAS/STING activation in tumors (29, 30).

Several studies have indicated that cancer-associated genomic instability can also modulate cGAS/STING signals. For example, BRAC1/2 promotes the repair of double-stranded DNA breaks (31). A statistical investigation pointed out that individuals with germline BRCA1/2 mutations have a higher risk of developing breast and ovarian cancer than bystanders (32). In such tumors, the cGAS-STING signaling pathway is activated due to the inability to cope with endogenous DNA damage or to follow exogenous DNA damage, resulting in massive lymphocytic infiltration (33). Interestingly, tumors with a BRCA1 mutation often carry amplifications in the MYC oncogene, and a recent study has shown that MYC can lead to immune escape by suppressing STING-dependent innate immunity (34). Besides, Neurofibromin 2 (NF2/Merlin/schwannomin) is a classical tumor suppressor and naturally occurring mutations in the FERM domain could transform it into a profound suppressor, which can inhibit the cGAS-STING pathway by blocking STING-mediated DNA sensing. This evidence may provide some clues to the pathogenesis of NF2-related tumors (35).

### 2.3 Noncanonical Activation of the cGAS/STING Signaling Pathway

Although micronucleus cGAS can detect DNA damage and induce immune responses, nuclear cGAS has recently been found to inhibit DNA repair in a STING non-dependent manner. In normal cells, homologous recombination’s accurate repair of DNA double-stranded breaks preserve genome integrity and inhibit tumorigenesis. However, stimulation of DNA damaging agents leads to dephosphorylation of cGAS. It facilitates its shuttling to the nucleus, where cGAS is recruited to the DNA double-strand break site and interacts with PARP1 *via* poly(ADP-ribose), hindering the formation of the PARP1-Timeless complex, thereby inhibiting homologous recombination and promoting tumorigenesis (36).

Moreover, STING can also regulate cell cycle in a cGAS-STING axis-independent manner in some tumor models. In human colon cancer cell HCT116, STING was observed to downregulate the expression of proliferation-related genes BUB1 (Budding Uninhibited by Benzimidazoles related 1) and MAD2L1 (Mitotic Arrest Deficient 2-like 1), indicating that STING may also play a role in tumor suppression in cancer cells that lack cGAS expression (37).

## 3 Role of cGAS/STING Pathway as a ‘Double-Edged Sword’ in Cancer

### 3.1 Antitumor Mechanisms of the cGAS/STING Pathway

**Figure 2:**
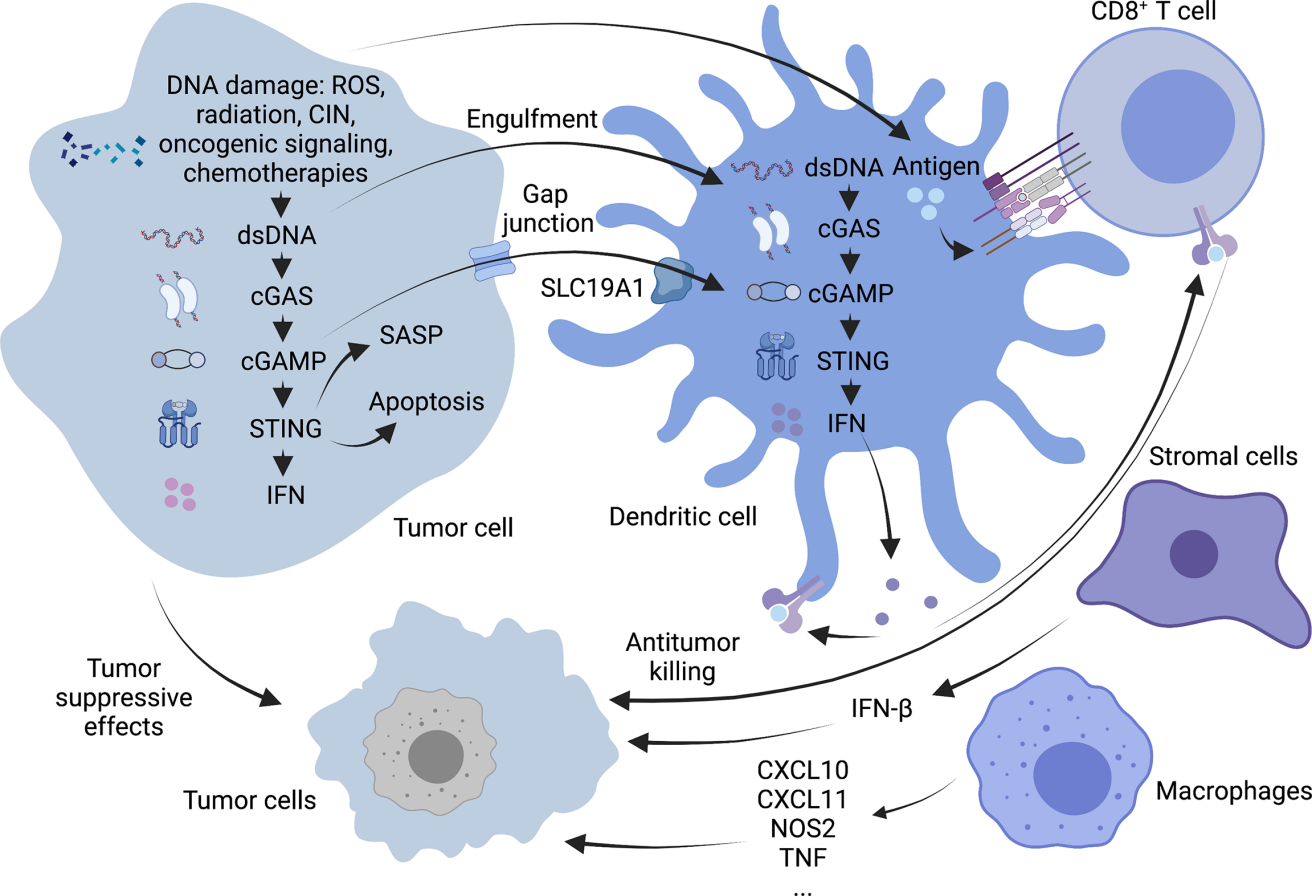
Antitumor mechanism of cGAS-STING pathway. cGAS-STING signaling exerts antitumor functions in cancer cells in an autonomous and non-autonomous manner. On the one hand, OIS secretes SASP in response to oncogenic stimuli to prevent cell proliferation, and activation of the STING signaling pathway in tumor cells may also promote apoptosis in tumor cells. On the other hand, STING activation in tumor cells promotes downstream type I IFN transcription and allows cross-talk between tumor and neighboring immune cells to modulate antitumor immunity. DCs can capture tumor-associated antigens and subsequently initiate tumor-killing by CD8^+^ T cells. Macrophages can produce TNF-α and express high levels of CxCl10, CxCl11, NOS2 and other molecules. Stromal cells can also be effective against tumor angiogenesis by expressing IFN-β. Created with BioRender.com.

cGAS/STING signaling plays an antitumor role in cancer cells in both an autonomous and non-autonomous manner (**Figure 2**). In the precancerous stage, oncogene-induced senescence signaling inhibits cancer by activating the p53 or p16-retinoblastoma protein pathway in response to carcinogenic stimuli, including carcinogenic stress, permanently inhibiting cell proliferation (38). It is characterized by the secretion of inflammatory mediators, consisting of various cytokines, chemokines, extracellular matrix proteins and growth factors, collectively known as the aging-related secretory phenotype (SASP) (39). Some studies have suggested that the cGAS/STING pathway regulates cell senescence mainly by suppressing soluble factors and inducing SASP autocrine and paracrine signal transduction. In turn, the rupture of the nuclear membrane and the appearance of cytoplasmic chromatin fragments (CCFs) caused by the loss of lamin B1 (40) in senescent cells can cause acute STING signal transduction and activation (41). The loss of ataxia telangiectasia-mutated (ATM) kinase leads to the accumulation of cytoplasmic DNA, which is partially released from the damaged mitochondria and triggers the phenotype of STING-dependent aging in the brain and *in vitro* (42). SASP may have both beneficial and disadvantageous effects. The reason for this is that some SASP components can maintain senescence stagnation through the autocrine cytokine network. When it exists for an extended period, the secretory activity may be harmful. Chronic SASP can induce epithelial-mesenchymal transformation and invasiveness through paracrine mechanisms dependent on the SASP factors IL-6 and IL-8 and the expression of RAS and the loss of function of p53 may significantly accelerate this phenomenon (43). The activation of the STING signaling pathway in cancer cells may also promote the apoptosis of tumor cells. When cells are affected by internal apoptosis-stimulating factors, such as the activation of oncogenes, DNA damage, hypoxia and the loss of cell growth factors, it can activate the mitochondrial apoptotic pathway, promoting apoptosis. In vertebrates, this mechanism contributes to mtDNA leakage and activation of the mtDNA-dependent cGAS-STING signaling pathway through mitochondrial outer membrane permeabilization (MOMP) induced by Bax and Bak, leading to I-IFN production. STING agonists can downregulate the anti-apoptotic protein BCL-2 and increase the proapoptotic factor Bax to drive apoptosis. It is worth noting that malignant T cells exhibit a high susceptibility to such death-induced characteristics of STING. Thus, STING agonists may help exert an antitumor effect on T cell malignant tumors (44). Besides, the activation of STING in tumor cells also promotes the transcription of downstream type I IFN, induces the maturation of dendritic cells (DCs) and recruits supportive immune cells to remove tumors (45).

In addition to tumor cells that can specifically activate the cGAS-STING pathway, some antigen-presenting cells (APCs), such as DCs and macrophages, can also initiate the body’s anti-tumor response *via* this pathway. In particular, activation of STING signaling in the DCs lineage driven by the basic leucine zipper transcription factor ATF-like 3 (BATF3) is a central step in the overall cancer immune cycle (46). Tumor cell DNA is a key inducer of innate immunity. It mainly includes tumor cell nuclear or mitochondrial DNA encapsulated in exosomes (47) and DNA released due to tumor cell turnover and death. Uptake of this DNA by the host APC activates the intracellular STING signaling pathway, leading to the secretion of type I interferons and chemokines, C-X-C motif chemokine ligands (CXCL)9 and CXCL10 (48). And then tumor cells with a T-cell inflammatory phenotype are phagocytosed by antigen-presenting cells which are activated by those cytokines mentioned above. Tumor-infiltrating Batf3 DCs take up tumor-associated antigens and migrate through lymphatic vessels to tumor-draining lymph nodes, where antigens are captured on major histocompatibility complex (MHC) I and MHC II molecules are presented to T cells and tumor-specific CD8^+^ T cells are cross-activated. Activated CD8^+^ T cells subsequently clone and expand in tumor-draining lymph nodes and kill cancer cells *via* vascular transport (49). Damaged cancer cells then release additional tumor-associated antigens (performing the first step again), increasing the breadth and depth of the response in subsequent cycles, which forms a positive feedback loop called the cancer-immune cycle (50). Although the role of intrinsic cGAS-STING activation in T cells is unknown, it has been shown that cGAS-STING-mediated type I interferon signaling enhances the stem cell-like CD8^+^ T cell differentiation program by inhibiting Akt activity (51). In addition, tumor cell-derived cGAMP can also transfer to host DCs through the folate transporter SLC19A1 and then directly bind to STING to activate it in DCs (52). CD8^+^ T cells can recognize target cells and eliminate them mainly *via* exocytosis-mediated apoptosis with granules containing the effector molecules, perforin and granzyme. Perforins form transmembrane pores in the cancer cells, allowing the extracellular environment (including granzymes) to directly diffuse into the cytoplasm, where they activate the apoptotic pathway through proteolytic substrate treatment (53).

Although DCs are considered the primary responders of tumor cGAMP (54), other cells, such as macrophages and stromal cells, may also have this function. In two tumor models that employed a mouse SCC cell line (mSCC1) that was generated from methylcholanthrene-induced carcinoma and a mouse CT26 colon cancer cell line, research shows that mature CD11b^mid^ Ly6C^+^ F4/80^+^ MHC class II^+^ macrophages can instantly migrate to the tumor site through a STING-dependent signaling pathway in the tumor microenvironment. All these macrophages have familiar characteristics, such as the production of TNF-α; expressing CXCL10, CXCL11, NOS2, and IFNB1 at high levels; exert phagocytic activity but cannot produce IL-10, suggesting that they are effective antitumor effector cells (55).

As vital members of the tumor microenvironment, Stromal cells (such as endothelial cells and fibroblasts etc.) also express the STING gene. It is generally considered that DCs are the primary source of type I IFN in the immune response process (56). However, studies have identified endothelial cells as the most important IFN-producing cells responding to the spontaneous and enforced STING activation (57). Of note, in mouse melanoma models, researchers have found that IFN-β is expressed exclusively in the tumor microenvironment but have not detected IFN-α. This result may be related to the weak ability of endothelial cells to produce IFN-α following STING activation (57). IFN-β is an effective anti-angiogenic cytokine. It can suppress the proliferation or survival of endothelial cells and inhibit the formation of the capillary network. It has been demonstrated that intratumoral STING agonist therapy can activate type I IFN signaling and can induce the upregulation of vascular normalizing genes in Lewis lung carcinoma (LLC) models, such as angiopoietin 1 (ANGPT1), platelet-derived growth factor receptor beta (PDGFRB), melanoma cell adhesion molecule (MCAM) and cadherin 5 (CDH5). These changes lead to increased pericyte coverage, structural normalization of tumor blood vessels, and the basement membrane’s integrity, thereafter promoting the intratumoral infiltration of effector CD8^+^ T cells and alleviating hypoxia in the tumor microenvironment (58).

Type I INFs play multiple roles in inducing antitumor immunity. IFN-α and IFN-β produced by cancer cells bind to IFN receptors on the same cell, adjacent cells, or immune cells and induce autocrine or paracrine signals. The latter promotes the mobilization of immune cells, such as DCs and T cells, to eradicate tumors. IFN-1 is considered to increase the number of DCs and stimulate the maturation of DCs in the tumor microenvironment following cGAMP injection (57). It can also reduce the rate of endosomal-lysosomal system acidification and promote the storage of exogenous antigens in DCs. In addition, IFN-α can promote the localization of MHC I molecules in the antigen storage area of DCs and enhance the antigen presentation ability of DCs. Furthermore, IFN-1 can promote the production of chemokines (such as CXCL9 and CXCL10) and then enable the homing of antigen-presenting cells and the migration of CD8^+^ T and natural killer cells (48). Finally, IFN-1 suppresses the immunosuppression of regulatory T- (Treg) cells by downregulating phosphodiesterase 4 (PDE4) and upregulating cyclic adenosine monophosphate (CAMP) (45).

### 3.2 Tumor-Promoting Mechanism of the cGAS/STING Pathway

Based on The Cancer Genome Atlas (TCGA) dataset containing 18 types of malignant tumors, some scholars have compared the changes in the expression of critical molecules in the cGAS/STING signaling pathway between malignant and normal control tissues, including MB21D1 encoding cGAS and TMEM173 encoding STING, as well as TBK1 and IRF3. It has been found that the expression of these four essential molecular genes is significantly upregulated in almost all detected cancer types, suggesting that cGAS/STING signal transduction may be activated in all cancers (49). With further in-depth research and the presentation of new evidence (36, 59), it is generally considered that the cGAS/STING signaling cascade may play a dichotomous role in the development of tumors. In some cases, highly invasive and unstable tumors can paradoxically use cGAS/STING signals to stimulate carcinogenesis (60).

**Figure 3:**
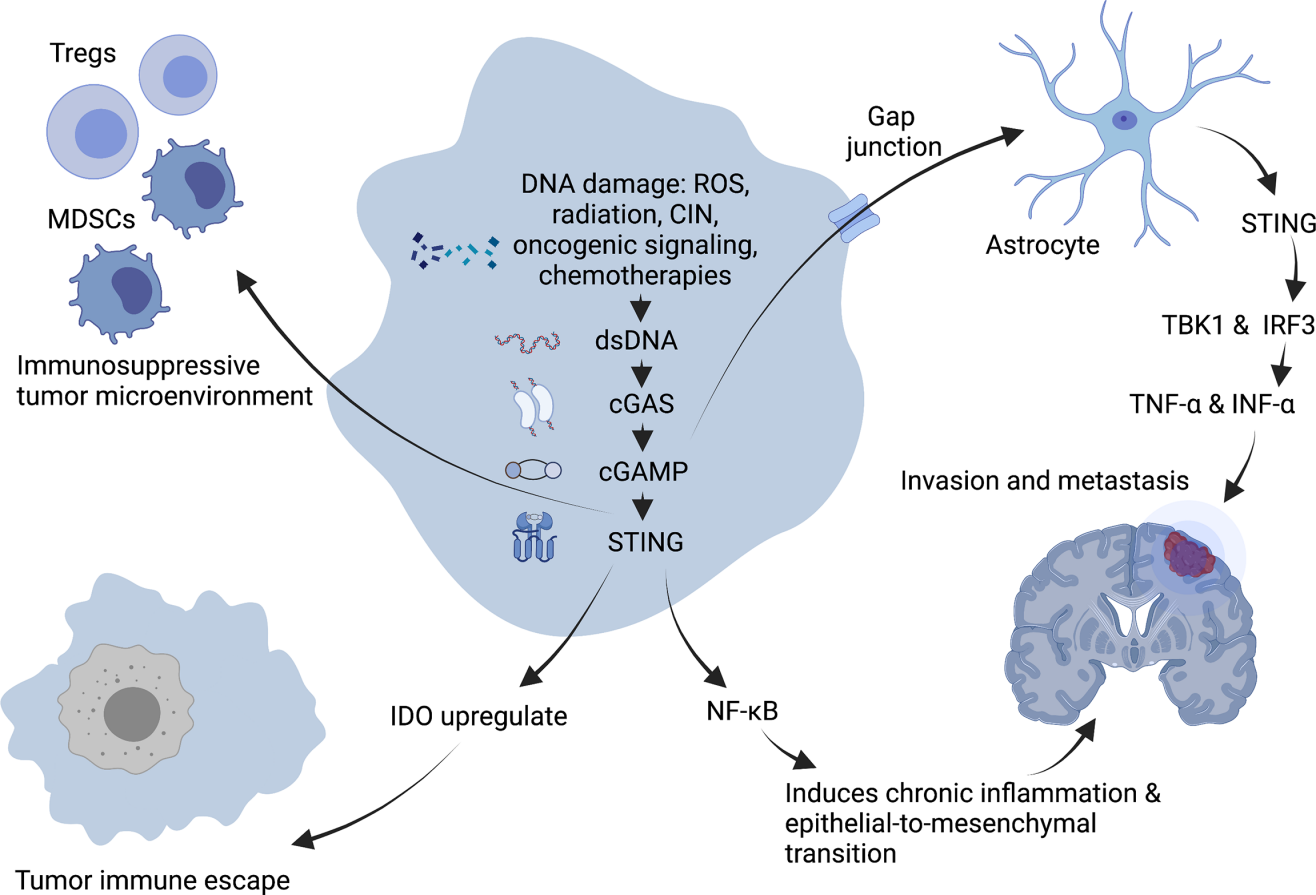
Tumor-promoting mechanism of cGAS-STING pathway. DNA damage induces chronic inflammatory signaling through sustained activation of NF-κB downstream of the cGAS-STING signaling pathway, facilitating epithelial-to-mesenchymal transition and leading to increased migration and invasion of cancer cells. Expression of STING in tumors promotes immunosuppressive cells such as Treg and MDSC to infiltrate into tumor sites, and upregulation of IDO expression promotes immune escape of tumors in the body, creating an immunosuppressive tumor microenvironment. In addition, the second messenger, cGAMP can be transferred through the cancer-astrocyte gap junction to activate STING protein in astrocytes, thus triggering TBK1 and IRF3 activation as well as IFNα and TNFα production to promote tumor brain metastasis. Created with BioRender.com.

As a critical activator of the inflammatory response, NF-κB participates in regulating cell survival, proliferation and apoptosis. In addition, NF- κB promotes the occurrence and development of inflammation, immune diseases and tumors (61). Chromosome instability (CIN) can induce chronic inflammatory signal transduction by continuously activating cGAS/STING signaling and the downstream NF-κB pathway, resulting in the increased migration and invasion of cancer cells (62) (**Figure 3**). For example, 7,12-dimethylbenz(a)anthracene, a carcinogen of skin cancer, can cause nuclear DNA leakage into the cytoplasm to activate the STING signaling pathway, resulting in the production of inflammatory cytokines and skin inflammation and ultimately inducing skin carcinogenesis (63). Furthermore, from TCGA database analysis, it has been demonstrated that the expression level of STING in tumors is negatively correlated with the infiltration of immune cells in some tumor types, which means a high level of cGAS/STING signaling may predict a poor prognosis in patients with some cancers (49).

A characteristic of an immunosuppressive tumor microenvironment is the upregulated expression of immune checkpoint indoleamine-2,3-dioxygenase (IDO). Existing study suggest that STING can upregulate IDO expression to directly or indirectly suppress T-cell numbers and function and then promoting immune immune escape (64).

The STING signal is closely related to tumor metastasis. CIN induces the production of a large number of micronuclei. When nuclear membrane breaks, genomic DNA will overflow into the cytoplasm, activating the cGAS/STING signaling pathway and triggering irregular NF-κB signals and inflammatory responses, conducive to the transformation of epithelial-to-mesenchymal transition. It has been demonstrated that cells with a high CIN have a higher migratory and invasive ability *in vitro* (59). Moreover, the metastasis of tumor cells may be driven by non-autonomous cGAS/STING pathway-related mechanisms (59). It has been demonstrated that some cancer cells can selectively promote the assembly of gap junctions between cancer cells and astrocytes composed of connexin 43 (Cx43) by expressing protocadherin 7 (PCDH7). These channels allow the transfer of the second messenger, cGAMP, from cancer cells to adjacent astrocytes to activate STING, thereby triggering TBK1 and IRF3 and producing IFN-α and TNF-α. As paracrine signals, these factors can further activate the STAT1 and NF-κB pathways in metastatic brain cells, finally promoting brain metastasis and chemotherapeutic resistance in breast and lung cancer (65).

### 3.3 The Multiple Functions of cGAMP

cGAMP plays a crucial role in cGAS-STING signaling by binding and activating STING, which has a greater potential for immunotherapy. However, the role played by cGAMP varies among different cells.

In myeloid cells, cGAMP agonists can activate their cGAS-STING signaling pathway, thereby promoting their killing of tumor cells. For example, transient accumulation of CD11b ^+^ Ly6C ^+^ F4/80 ^+^ monocytes/macrophages at the tumor site by STING stimulation can be observed after cGAMP injection, enhance the phagocytic activity of cells for antitumor purpose (55). However, in tumor cells, cGAMP can play two quite different roles. In early preneoplastic cells, the cGAS-STING pathway acts as a tumor suppressor. Tumor-derived cGAMP can initiate antitumor immunity *via* downstream canonical NF-κB signaling pathway (66, 67). It can also activate cGAS-STING signaling through antigen-presenting cell, same as tumor DNA, to trigger immune cell-mediated tumor clearance. For instance, activated cGAMP within tumor cells can be transferred to host cells to trigger STING and subsequently activate the anti-tumor response of NK cells (68). In late tumor stages, sustained cGAMP expression by tumor cells leads to activation of downstream non-canonical NF-κB signaling pathways, releasing other pro-inflammatory cytokines such as IDO, thus creating an immunosuppressive tumor microenvironment that promotes tumor progression (69).

Overall, the impact of cGAMP on cancer depends on the period and type of tumorigenesis and the activated cell type, among others. This requires additional exploration to determine its exact role in oncology.

## 4 STING and Autophagy in Cancer

Cellular autophagy is a lysosome-based intracellular degradation process that aims to maintain the organism’s essential cell survival and homeostasis under various cellular stress conditions such as hypoxia and infection. In cells, activating the autophagy-related 1/autophagy activating kinase 1 (ATG1/ULK1) complex is essential. They can phosphorylate or recruit their downstream proteins to promote autophagosome formation and thus induce typical autophagy (70, 71). However, it was recently found that after stimulation of cGAMP production by cGAS from dsDNA generated by various causes of damage, STING can translocate to the endoplasmic reticulum-Golgi intermediate compartment (ERGIC), which could serve as a membrane source for LC3 lipidation. cGAMP can induce LC3 lipidation by relying on WIPI2 and ATG, 5 but not ULK and Vps34-Beclin kinase complexes. Subsequently, LC3-positive membranes wrap dsDNA, bacteria, or viruses and form autophagosomes, suggesting that STING can also activate atypical autophagy through a mechanism independent of interferon induction (72).

Similar to STING, autophagy plays a bidirectional role in tumor progression. A terminal response called replicative crisis occurs during early tumorigenesis, which leads to delayed mitosis, telomere deprotection amplification and cell death. Telomeric DNA damage activates the cGAS-STING pathway. The researchers found that cells with cGAS or STING removed bypassed the crisis to proliferate and exhibited a reduction in LC3-II and accumulation of P62 in the cells (73). This evidence suggests that STING-mediated autophagy may be an additional barrier to early tumor progression in normal cells. However, the endoplasmic reticulum stress response and autophagy may also allow cancer cells to survive in a stressful environment, thereby promoting the development of advanced cancer (74).

## 5 STING Agonists in Cancer Therapy

### 5.1 Overview of STING Agonists

As the activation of the cGAS/STING pathway in the tumor microenvironment can induce the effective cross-priming of tumor-specific antigens and promote the infiltration of effector T cells (45), STING agonists have been developed to simulate this activation in order to enhance the anticancer effect. Thus far, several types of stimulants have been found, mainly divided into three categories: Cyclic dinucleotides (CDNs) and their derivatives, DMXAA and its analogs, and small molecule agonists.

CDNs are divided into several CDN families, which are composed of cyclic di-GMP (c-di-GMP), cyclic di-AMP (c-di-AMP), and cyclic AMP-GMP (cGAMP) molecules. cGAMP includes 3’,3’-cGAMP, 2’,3’-cGAMP, 3’,5’-cGAMP and 2’,5’-cGAMP (75). Their antitumor regulatory effect was first found in c-di-GMP. c-di-GMP was initially regarded as an allosteric regulator of cellulose synthase in *Gluconacetobacter xylinus* (formerly known as *Acetobacter xylinum*) and is currently considered to be a ubiquitous second messenger of bacteria (76). The injection of c-di-GMP into a newborn glioma model can improve the survival rate of glioma-bearing mice in a STING-dependent manner, such as enhancing the signal of IFN-1, CXCL10, and the migration of T cells to the brain (77). c-di-GMP can also combat metastatic breast cancer *via* numerous methods. A mouse metastatic breast cancer model observed that a low dose of c-di-GMP provided a potent adjuvant effect with the LM-Mb vaccine by promoting the production of IL-12 derived from MDSCs and increasing the response of CD8^+^ T cells to the tumor-associated antigen Mage-b. Moreover, a large dose of c-di-GMP can activate caspase-3 and kill cancer cells directly (78). 2’,3’-cGAMP is the second messenger of STING signal transduction in mammalian cells. In murine colon 26 adenocarcinoma, cGAMP inhibited tumor growth mainly by activating STING and its downstream STING-IRF3 signaling. Analysis of host serum and tumor tissues showed that cGAMP significantly upregulated anti-tumor cytokines such as IFN-β and IFN-γ and activated DCs. Interestingly, cGAMP still had some tumor-suppressive activity in STING-/- mice, suggesting that cGAMP may stimulate other STING-independent pathways to suppress tumor when STING is not present growth (79). A study using a model of mouse cervical cancer demonstrated that the use of the mutant type of HPV 16 E7 protein (E7GRG) as a therapeutic vaccine candidate antigen-stimulated the cell-mediated and humoral immune response and inhibited tumor growth. The co-administration of 2’,3’-cGAMP, E7GRG and CpG-C adjuvant exerted a synergistic effect, establishing a shift towards the Th1 type immune response and decreasing tumor growth (80). In malignant B-cell tumors, 3’3’-cGAMP induces the prolonged presence of STING in the endoplasmic reticulum or Golgi apparatus to form protein complexes to activate apoptosis, without any significant association with activated IFN. This suggests that STING agonists have potential therapeutic use for the treatment of B-cell malignancies such as chronic lymphatic leukemia, multiple myeloma, etc., in addition to their immunomodulatory activity against cancer (81). It was also shown that 2’,5’-cGAMP showed stronger complex formation with both human and mouse STING compared to 3’,5’-cGAMP and was unaffected by the species specificity of the bite, just like DNA. Moreover, cGAS-dependent two-step synthetic 2’,5’-cGAMP can be used to develop specific inhibitors to treat autoimmune diseases related to cGAS/STING signaling (82).

Based on natural CDNs, synthetic CDNs have been developed with optimal performance. ADU-S100 (ML RRS2 CDA or MIW815) was the first STING agonist used in human clinical trials for cancer immunotherapy. Compared with other CDNs, ADU-S100 exhibits higher stability and lipophilicity. It has been indicated that in B16 melanoma, CT26 colon cancer, or 4T1 breast cancer models, the injection of ADU-S100 effectively triggers CD8^+^ T cell responses in a STING-dependent manner and leads to significant tumor regression, exerting long-lasting antitumor effects (83). Intraperitoneal injection of ADU-S100 into mouse models with peritoneal carcinomatosis of ovarian cancer (ID8) and MC38 colon carcinoma observed inhibition of peritoneal carcinomatosis and malignant ascites progression.ADU-100 effectively reduced abnormal tumor vasculature formation by activating STING-mediated type I IFN and upregulated the number of CD8^+^ T cells, enhancing the remaining tumor vasculature of pericyte coverage, thereby inhibiting the formation of malignant ascites in the peritoneal cavity. During peritoneal metastasis, type I IFN signaling also inhibited the recruitment of CD206^+^ M2-like macrophages (84). Notably, the intratumoral injection of ADU-S100 was shown to be well-tolerated in patients with advanced solid tumors and lymphomas, and no DLT was reported (85). IACS-8779 and IACS-8803 are highly efficient CDN STING agonists that introduce a group of reasonably selected and targeted modifications into the nucleobase and ribose sections of the 2’,3’ CDA structure. These two drugs manifest a superior ability to activate the STING pathway *in vitro* and a more prominent systemic antitumor response than the clinical benchmark agonist, ADU-S100, in a B16 murine model of melanoma (86). MK-1454 is a synthetic CDN analog developed by Merck to treat advanced/metastatic solid tumors or lymphomas. The preliminary results showed good activity and safety in a study of the drug as a monotherapy and combined with pembrolizumab entering a phase I clinical trial. PRs observed in head and neck squamous cell carcinoma, triple-negative breast cancer and undifferentiated thyroid cancer (87).

Flavone 8-acetic acid (FAA) was initially synthesized as a non-steroidal anti-inflammatory drug (88). It induces extensive hemorrhagic necrosis associated with inhibiting tumor blood flow (89) and has exhibited significant antitumor activity in preclinical studies (90). However, the results obtained for FAA in clinical trials were not positive (91). Thus, a more active FAA analog, 5,6-dimethylxanthenone-4-acetic acid (DMXAA), was developed. DMXAA, also known as ASA404 and Vadimezan, can fight tumors directly and indirectly. First of all, DMXAA can induce apoptosis in tumor endothelial cells, increase specificity, and irreversibly directly destroy the established tumor vascular system and completely block the tumor blood flow. Secondly, DMXAA can indirectly activate the innate immune system and can induce the production of inflammatory cytokines. Clinical trials have been better results in selected patients with melanoma and squamous cell carcinoma (88).

At present, amidobenzimidazole (ABZI) represents a breakthrough for STING agonists in cancer treatment. By replacing its N1-hydroxyphenethyl moiety with a 4-carbon butane linker, researchers have derived a compound named diABZI. It has been demonstrated that the binding affinity of this new compound is significantly enhanced, and *in vivo*, it can lead to the adaptive response of CD8^+^ T cells. The intravenous administration of diABZI STING agonist to mice with a normal immune function and with established homologous colon tumors has been shown to exert potent antitumor effects, diminishing the tumor completely and persistently. The diABZI compound is the first effective non-nucleotide STING agonist used globally and has immense potential to improve the immunotherapy of human cancers (92).

### 5.2 Combination of STING Agonists With Other Types of Cancer Treatment

#### 5.2.1 STING Agonists and Classical Cancer Therapies

Classical therapies, such as radiotherapy and chemotherapy, are the main methods for treating human cancers (93). Radiation-induced DNA damage leads to micronuclei formation or cell death and can activate immune cells’ cGAS/STING pathway (54). For example, exposure to gamma-rays can directly induce the production of type III IFN (mainly IFNL1) in human cancer cell lines through the cGAS/STING/TBK1/IRF1 signaling pathway (94). However, radiation may also exert an adverse inhibitory effect on the immune system. It has been demonstrated that radiation can activate the immunosuppressive TGF-β cytokine (95) and can promote the accumulation of Tregs (96) and pro-tumorigenic M2 macrophages (97). cGAS is activated by radiation in a dose-dependent manner. Evidence indicates that when the radiation dose exceeds 12-18 Gy, DNA exonuclease TREX1 helps degrade the DNA accumulated in the cytoplasm to attenuate its immunogenicity and downregulate the cGAS pathway. Conversely, radiation repeatedly below the threshold dose for the induction of TREX1 stimulates IFN-β in cancer cells (98). Chemotherapy can induce DNA damage and inhibit DNA repair simultaneously. The damaged DNA activates the cGAS/STING axis to enhance DC-mediated antigen presentation and T cell response (45). For example, one of the mechanisms of cisplatin therapy is to initiate the cGAS/STING pathway *via* the upregulation of the protein levels of cGAS and STING (99). In addition, the anthracycline antibiotics, adriamycin and daunorubicin, which can inhibit topoisomerase II, induce an IFN response and trigger the cGAS/STING pathway in an ATM kinase-dependent manner (100). Although radiotherapy and chemotherapy do not directly target the cGAS pathway, these treatments can activate the cGAS/STING axis in a ‘roundabout’ manner and can enhance the antitumor immune response. The co-administration of STING agonists with radiotherapy or chemical drugs can enhance the antitumor effect and reduce the side-effects caused by classical therapies. It has been demonstrated that the inhalation of phosphatidylserine-coated liposomes loaded with cGAMP (NP-cGAMP), a STING agonist, in mouse models of lung metastases can rapidly distribute NP-cGAMP to the lungs and stimulate STING signal transduction in antigen-presenting cells, promoting the production of type I IFN, and the synergistic effect of NP-cGAMP and graded IR (8 Gy x 3) can suppress tumor metastasis at the site of radiotherapy and lung metastasis without radiotherapy (101). 5-Fluorouracil (5-FU) is an effective clinical anti-neoplastic drug that exerts its effects by interfering with DNA synthesis; however, it also has noticeable side effects, such as nausea, mucosal necrosis, and hematochezia. When used in combination with cGAMP, a stimulant of innate immune antitumor agents, the antitumor effect of 5-FU is enhanced, while the side effects are reduced (79).

#### 5.2.2 STING Agonists and Immune Checkpoint Inhibitors

T cells are the primary therapeutic focus of endogenous antitumor immunity due to their ability to selectively recognize peptides from proteins in all cell compartments, directly recognize and kill cells expressing specific antigens, and coordinate various immune responses with the help of CD4^+^ helper T cells. Therefore, specific targeting agonists of co-stimulatory receptors or antagonists of inhibitory signals, such as targeting cytotoxic T lymphocyte-associated antigen 4 (CTLA4) and programmed cell death protein 1 (PD1), can be leading to the enhancement of the antigen-specific T cell response (102–104). The activation of STING increases the critical Th1-recruiting cytokines, CXCL9 and CXCL10, and IFN-1. Type I IFN can promote the activation and maturation of DCs, improve the antigen presentation of CD4^+^ T lymphocytes and enhance antigen cross-presentation to CD8^+^ T lymphocytes, hence, T cell priming. Therefore, STING agonists seem to be ideal partners for immune checkpoint inhibitors (105). Compared with no treatment or treatment with immune-checkpoint inhibitors alone, the co-administration of STING agonists with CTLA4 and PD1 antibodies in a preclinical HPV^+^ oral tumor model displayed the most significant survival advantage (106). Moreover, in B16F10- and BRAF-mutated murine models, the group treated with cGAMP encapsulated into lipid nanoparticles conjugated with mannose (LP-cGAMP) and anti-programmed death-ligand 1 (PD-L1) exhibited a more long-lasting inhibition of tumor growth and achieved a more prolonged survival than others (107).

#### 5.2.3 STING Agonists and Tumor Vaccine

Due to central and peripheral tolerance, the immunogenicity of tumor-associated antigens is not potent enough to trigger a firm tumor antigen-specific CD8^+^ T cell response, which is essential for effective antitumor immunity (108). Therefore, appropriate adjuvants play an indispensable role in overcoming tolerance and enhancing tumor-specific immune responses. STINGVAX is the first designed STING-based tumor vaccine containing granulocyte-macrophage colony-stimulating factor (GM-CSF) and CDN ligands. In multiple syngeneic mouse models, the *in vivo* antitumor responses for STINGVAX were more prominent than those for the GM-vaccine. STINGVAX-treated mice exhibited increased tumor-infiltrating lymphocytes (TILs) and an upregulated PD-L1 expression, indicating that STINGVAX is suitable for co-administration with anti-PD-1 therapy (109). In a previously mentioned study using a metastatic breast cancer mouse model, the researchers used a *Listeria* monocytogenes (LM)-based vaccine with the expression of tumor-associated antigen Mage-b (LM-Mb) as the vaccine and c-di-GMP as the stimulating ligand. This model observed the enhanced presentation of tumor-associated antigens and increased tumor-associated antigen-specific T cell activation *in vivo*. The combination of c-di-GMP and LM-Mb also significantly reduced the number of MDSCs in the blood. The reduction in the MDSC population helps to reduce immunosuppression in the tumor microenvironment, reducing the growth of tumors and metastases. As MDSCs and immunosuppression exist in almost all cancers, using c-di-GMP as an adjuvant against other cancers is promising (78).

#### 5.2.4 STING Agonists and Chimeric Antigen Receptor (CAR) T Cell Therapy

CAR T cell therapy is one of the most promising methods for anticancer treatment. T cells are redirected against a tumor following the enforced expression of CARs. CAR, as the core component of CAR T cells, enables T cells to recognize tumor antigens in an HLA-independent manner and identify a broader range of target antigens than natural T cell surface receptor (TCR) (110–112). In an orthotopic murine breast tumor model, a previous study proved that Th/Tc17 CAR T cells persist durably in the tumor microenvironment and play a prominent role in tumor growth control. However, the number of Th/Tc17 CAR T cells is only moderately increased in the tumor microenvironment, improving early tumor suppression but not leading to a long-term antitumor response. The study also compared the T cell population between two CAR T cell treatment groups combined with or without DMAXX. The results revealed that the combined treatment group expressed higher CXCL9 and CXCL10, which could recruit CXCR3-expressing Th/Tc17 T cells, leading to a more significant number of CAR T cells (113).

#### 5.2.5 STING Agonists and Oncolytic Virus (OV)

OV plays its antitumor role mainly *via* both direct and indirect mechanisms. On the one hand, it replicates in and lyses tumor cells selectively. On the other hand, OV acts as an ‘*in situ’* vaccine and directs the host immune response to target tumors. Upon the lysis of tumor cells, viral offspring are released to infect neighboring cancer cells and finally induce the systemic antitumor response (114). In 2015, the US Food and Drug Administration (FDA) approved herpesvirus talimogene laherparepvec (T-VEC) for advanced melanoma, rendering it the first OV to be approved in the United States (115). Patients with metastatic melanoma present a high level of Tregs, Ts cells, and MDSCs within established tumors. Following the injection of Oncovex^GM-CSF^, an oncolytic herpes simplex virus engineered to express GM-CSF, the researchers observed a decreased level of these suppressor cell populations and the production of local and systemic MART-1-specific CD8^+^ effector cells. which suggests that Oncovex^GM-CSF^ can induce potent local and systemic T cell immunity (116). The enhanced replication and intercellular spread of cancer cells due to STING deficiency make the effectiveness of oncolytic therapy in certain tumors can be significantly increased. For example, STING-defective melanoma cells are highly sensitive to DNA virus-mediated oncolytic effect. Therefore, it is now widely considered that STING/cGAS may be a more effective biomarker for prognosis (117).

## 6 STING Inhibitors in Cancer Therapy

Dysfunctional nucleases and abnormal mutations in STING can lead to abnormal activation of the cGAS-STING pathway, resulting in increased expression of IFN. Therefore STING inhibitors may be developed for use in a variety of interferon diseases. The existing inhibitors can be broadly classified into two categories, namely competitive antagonists that occupy CDN binding sites and inhibitors that covalently modify Csy88 and Csy91 to inhibit palmitoylation (118).

### 6.1 STING Antagonists Targeting the CDN-Binding Site

Based on biophysical and X-ray crystallography data, the STING C-terminal domain exists as a symmetric dimer with the ligand-binding site located at the interface between the two monomers, which is the binding site for the agonist CDN. Before binding to cGAMP, STING apolipoprotein adopts an “open” conformation, compared to a “closed” conformation after binding. It suggests that the “open” conformation may lead to inactivation. While the “closed” conformation may lead to protein activation. Tetrahydroisoquinolone can bind to the cGAMP binding site in the inactive “open” conformation. By taking advantage of the natural symmetry of the STING protein and utilizing a 2:1 binding stoichiometry, these compounds can fully occupy the binding pocket while mitigating the undesirable physicochemical properties associated with larger ligands. After incubation of THP1 cells with Tetrahydroisoquinolone, it was observed that this compound did not stimulate interferoβproduction and moderately inhibited cGAMP-induced interferonβproduction (119).

Astin C is a cyclic peptide isolated from the medicinal plant Aster tartaric that specifically targets STING and competes with CDN to bind the C-terminal activation pocket of STING. The exact mechanism of STING inhibition by astin C is still not fully determined, but it is known that this compound selectively inhibits the recruitment and activation of downstream IRF3 and does not affect the phosphorylation of STING and TBK1. It was demonstrated that astin C significantly attenuates the auto-inflammatory response in Trex1^-/-^ bone marrow-derived macrophages (BMDM) and disease models while having a low cytotoxic side effect in cellular and animal models. This suggests that astin C may be used in STING-mediated cancer and autoimmune diseases (120).

### 6.2 STING Antagonists Targeting STING Palmitoylation Sites

As described above, Csy88 and Csy91 in the cGAS-STING pathway induce STING palmitoylation. Allowing their subsequent assembly into multimeric complexes at the Golgi as well as recruitment of downstream signaling factors.

Protein mass spectrometry studies have shown that the 3-acylamino indole derivative Indole ureas (H-151) can form covalent bonds with Cys91. Intraperitoneal H-151 administration was performed in a mouse model to which a specific STING agonist was applied. The investigators observed that H-151 achieved effective systemic levels and showed a short half-life significantly reduced serum levels of type I IFN and IL-6 in mice. This suggests that H-151 is a potent small molecule antagonist of STING that can effectively inhibit type I IFN response, TBK1 phosphorylation and hsSTING palmitoylation (121).

Nitro fatty acids (NO2-FAs) are formed in response to HSV-2 infection. It was shown that NO2-FAs could directly modify STING by nitroalkylation at Cys88 and Csy91 and two adjacent cysteines of the N-terminal histidine (His16).Pretreatment with NO2 -FA substantially reduced the induction of type I interferon in response to HSV-2 in both THP-1 cells. Treatment with NO2-FA after HSV-2 infection also reduced the release of the IFN-induced cytokine CXCL10. In addition, NO2-FA treatment with STING-associated vasculopathy with onset in infancy (SAVI) also resulted in a reduced STING-dependent type I interferon response in immortalized fibroblasts from patients. Therefore, it is suggested that Nitro fatty acids can target STING signaling and reduce type I interferon release in mice and human cells and may be used in the future to treat STING-dependent inflammatory diseases (122).

## 7 Conclusion

By detecting micronuclei or cytoplasmic chromatin fragments induced by DNA damage, the cGAS/STING pathway can mediate the interplay between cytotoxic effects and immune stimulation, exerting a dichotomous effect on tumor tissue. However, the underlying mechanisms responsible for this phenomenon are still poorly understood and require further research. Therefore, further research is required to obtain a more in-depth knowledge of the STING-related tumor microenvironment and biological mechanisms to reveal the potential mechanisms of the STING-mediated immune response, such as the effects of the canonical and non-canonical NF-κB pathway on tumors. Furthermore, exploring the molecular details of the cGAS/STING pathway may enhance the current understanding of the antitumor mechanism of innate immunity and may provide a theoretical basis for future drug design for the treatment of tumors. Thus, this pathway may play a more critical role in basic immunology, tumor biology, and clinical tumor therapy.

The co-administration of classical therapies and STING agonists has demonstrated an excellent synergistic antitumor effect in several preclinical tumor models. These agonists can reduce the side-effects of drug use, overcome the tolerance to tumor treatments and achieve an optimal therapeutic effect, thus rendering the development of a new variety of antitumor treatments promising. Therefore, the comparison of the effects of various combination therapies and the exploration of their mechanisms is of utmost significance. Likewise, the current exploration of STING inhibitors provides valuable clues for further clinical drug development in the future.

## Author Contributions

YG performed the analyses and wrote the manuscript; XL, SH contributed to the conception of the paper; QL, XM contributed to analysis; PR, WW, WL helped perform the analysis with constructive discussions. All authors contributed to the article and approved the submitted version.

## Funding

This work was supported by the National Natural Science Foundation of China (No. 81972837, 82071986, 81771827, 81971721).

## Conflict of Interest

The authors declare that the research was conducted in the absence of any commercial or financial relationships that could be construed as a potential conflict of interest.

## Publisher’s Note

All claims expressed in this article are solely those of the authors and do not necessarily represent those of their affiliated organizations, or those of the publisher, the editors and the reviewers. Any product that may be evaluated in this article, or claim that may be made by its manufacturer, is not guaranteed or endorsed by the publisher.

## Glossary

**Table:**

cGAS	cyclic GMP-AMP synthase
STING	stimulator of interferon genes
cGAMP	Cyclic GMP-AMP
IFN	interferon
dsDNA	double-stranded DNA
LLPS	liquid-liquid phase separation
TREX1	three-prime repair exonuclease 1
ER	endoplasmic reticulum
TBK1	TANK-binding kinase 1
IRF3	interferon regulatory factor 3
JAK1	Janus kinase 1
STAT	signal transducer and activator of transcription
ISGs	interferon-stimulated genes
HCMV	human cytomegalovirus
HSV-1	Herpes simplex virus type 1
mtDNA	mitochondrial DNA
dsRNA	double-stranded RNA
DDR	DNA damage response
MLH1	MutL homolog 1
IR	ionizing radiation
DSB	DNA double-strand breaks
mtDNA	mitochondrial DNA
NF2	Neurofibromin 2
PARP-1	Poly [ADP-ribose] polymerase 1
BUB1	Budding Uninhibited by Benzimidazoles related 1
MAD2L1	Mitotic Arrest Deficient 2-like 1
SASP	senescence-associated secretory phenotype
CCFs	cytoplasmic chromatin fragments
DCs	dendritic cells
APCs	antigen-presenting cells
BATF3	basic leucine zipper transcription factor ATF-like 3
CXCL	C-X-C motif chemokine ligand
MHC	major histocompatibility complex
TNF-α	Tumor necrosis factor alpha
NOS	nitric oxide synthase
ANGPT1	angiopoietin 1
PDGFRB	platelet derived growth factor receptor beta
MCAM	melanoma cell adhesion molecule
CDH5	cadherin 5
Treg	regulatory T cells
PDE4	phosphodiesterase 4
CAMP	cyclic adenosine monophosphate
TCGA	The Cancer Genome Atlas
CIN	Chromosome instability
MDSC	myeloid-derived suppressor cell
IDO	indoleamine-2,3-dioxygenase
LLC	Lewis lung carcinoma
Cx43	connexin 43
PCDH7	protocadherin 7
ATG1/ULK1	autophagy related 1/autophagy activating kinase 1
ERGIC	endoplasmic reticulum-Golgi intermediate compartment
CDNs	cyclic dinucleotides
c-di-GMP	3’,5’-cyclic diguanylic acid
c-di-AMP	cyclic double AMP
OVA	ovalbumin
FAA	Flavone 8-acetic acid
DMXAA	5,6-dimethylxanthenone-4-acetic acid
ABZI	amidobenzimidazole
TGF-β	transforming growth factor-β
NP-cGAMP	phosphatidylserine-coated liposomes loaded with cGAMP
5-FU	5-Fluorouracil
CTLA4	cytotoxic T lymphocyte-associated antigen 4
PD1	programmed cell death protein 1
PD-L1	programmed death-ligand 1
TILs	tumor-infiltrating lymphocytes
LM	Listeria monocytogenes
CAR	chimeric antigen receptor
TCR	T cell surface receptor
OV	oncolytic virus
FDA	Food and Drug Administration
T-VEC	herpesvirus talimogene laherparepvec
BMDM	bone marrow-derived macrophages
SAVI	STING-associated vasculopathy with onset in infancy

## References

[1] Yum S Li M Chen ZJ. Old Dogs, New Trick: Classic Cancer Therapies Activate cGAS. Cell Res (2020) 30(8):639–48. doi: 10.1038/s41422-020-0346-1 PMC7395767 32541866

[2] Makkouk A Weiner GJ. Cancer Immunotherapy and Breaking Immune Tolerance: New Approaches to an Old Challenge. Cancer Res (2015) 75(1):5–10. doi: 10.1158/0008-5472.CAN-14-2538 25524899 PMC4286422

[3] Chen Q Sun L Chen ZJ. Regulation and Function of the cGAS-STING Pathway of Cytosolic DNA Sensing. Nat Immunol (2016) 17(10):1142–9. doi: 10.1038/ni.3558 27648547

[4] Sun L Wu J Du F Chen X Chen ZJ. Cyclic GMP-AMP Synthase Is a Cytosolic DNA Sensor That Activates the Type I Interferon Pathway. Science (New York NY) (2013) 339(6121):786–91. doi: 10.1126/science.1232458 PMC3863629 23258413

[5] Zhang X Wu J Du F Xu H Sun L Chen Z. The Cytosolic DNA Sensor cGAS Forms an Oligomeric Complex With DNA and Undergoes Switch-Like Conformational Changes in the Activation Loop. Cell Rep (2014) 6(3):421–30. doi: 10.1016/j.celrep.2014.01.003 PMC3969844 24462292

[6] Gao P Ascano M Wu Y Barchet W Gaffney BL Zillinger T. Cyclic [G(2',5')pA(3',5')p] is the Metazoan Second Messenger Produced by DNA-Activated Cyclic GMP-AMP Synthase. Cell (2013) 153(5):1094–107. doi: 10.1016/j.cell.2013.04.046 PMC4382009 23647843

[7] Xie W Lama L Adura C Tomita D Glickman JF Tuschl T. Human cGAS Catalytic Domain has an Additional DNA-Binding Interface That Enhances Enzymatic Activity and Liquid-Phase Condensation. Proc Natl Acad Sci USA (2019) 116(24):11946–55. doi: 10.1073/pnas.1905013116 PMC6575157 31142647

[8] Yu X Zhang L Shen J Zhai Y Jiang Q Yi M. The STING Phase-Separator Suppresses Innate Immune Signalling. Nat Cell Biol (2021) 23(4):330–40. doi: 10.1038/s41556-021-00659-0 33833429

[9] Ishikawa H Barber GN. STING Is an Endoplasmic Reticulum Adaptor That Facilitates Innate Immune Signalling. Nature (2008) 455(7213):674–8. doi: 10.1038/nature07317 PMC2804933 18724357

[10] Srikanth S Woo JS Wu B El-Sherbiny YM Leung J Chupradit K. The Ca Sensor STIM1 Regulates the Type I Interferon Response by Retaining the Signaling Adaptor STING at the Endoplasmic Reticulum. Nat Immunol (2019) 20(2):152–62. doi: 10.1038/s41590-018-0287-8 PMC6340781 30643259

[11] Mukai K Konno H Akiba T Uemura T Waguri S Kobayashi T. Activation of STING Requires Palmitoylation at the Golgi. Nat Commun (2016) 7:11932. doi: 10.1038/ncomms11932 27324217 PMC4919521

[12] Agalioti T Lomvardas S Parekh B Yie J Maniatis T Thanos D. Ordered Recruitment of Chromatin Modifying and General Transcription Factors to the IFN-Beta Promoter. Cell (2000) 103(4):667–78. doi: 10.1016/s0092-8674(00)00169-0 11106736

[13] Schneider WM Chevillotte MD Rice CM. Interferon-Stimulated Genes: A Complex Web of Host Defenses. Annu Rev Immunol (2014) 32:513–45. doi: 10.1146/annurev-immunol-032713-120231 PMC4313732 24555472

[14] West AP Khoury-Hanold W Staron M Tal MC Pineda CM Lang SM. Mitochondrial DNA Stress Primes the Antiviral Innate Immune Response. Nature (2015) 520(7548):553–7. doi: 10.1038/nature14156 PMC4409480 25642965

[15] Aguirre S Luthra P Sanchez-Aparicio MT Maestre AM Patel J Lamothe F. Dengue Virus NS2B Protein Targets cGAS for Degradation and Prevents Mitochondrial DNA Sensing During Infection. Nat Microbiol (2017) 2:17037. doi: 10.1038/nmicrobiol.2017.37 28346446 PMC7457382

[16] Liu H Golji J Brodeur LK Chung FS Chen JT deBeaumont RS. Tumor-Derived IFN Triggers Chronic Pathway Agonism and Sensitivity to ADAR Loss. Nat Med (2019) 25(1):95–102. doi: 10.1038/s41591-018-0302-5 30559422

[17] d'Adda di Fagagna F Reaper PM Clay-Farrace L Fiegler H Carr P Von Zglinicki T. A DNA Damage Checkpoint Response in Telomere-Initiated Senescence. Nature (2003) 426(6963):194–8. doi: 10.1038/nature02118 14608368

[18] Fenech M Kirsch-Volders M Natarajan AT Surralles J Crott JW Parry J. Molecular Mechanisms of Micronucleus, Nucleoplasmic Bridge and Nuclear Bud Formation in Mammalian and Human Cells. Mutagenesis (2011) 26(1):125–32. doi: 10.1093/mutage/geq052 21164193

[19] Hatch EM Fischer AH Deerinck TJ Hetzer MW. Catastrophic Nuclear Envelope Collapse in Cancer Cell Micronuclei. Cell (2013) 154(1):47–60. doi: 10.1016/j.cell.2013.06.007 23827674 PMC3749778

[20] Harding SM Benci JL Irianto J Discher DE Minn AJ Greenberg RA. Mitotic Progression Following DNA Damage Enables Pattern Recognition Within Micronuclei. Nature (2017) 548(7668):466–70. doi: 10.1038/nature23470 PMC5857357 28759889

[21] Li T Huang T Du M Chen X Du F Ren J. Phosphorylation and Chromatin Tethering Prevent cGAS Activation During Mitosis. Science (New York NY) (2021) 371(6535):eabc5386. doi: 10.1126/science.abc5386 PMC8171060 33542149

[22] Hanahan D Weinberg RA. Hallmarks of Cancer: The Next Generation. Cell (2011) 144(5):646–74. doi: 10.1016/j.cell.2011.02.013 21376230

[23] Luo J Solimini NL Elledge SJ. Principles of Cancer Therapy: Oncogene and non-Oncogene Addiction. Cell (2009) 136(5):823–37. doi: 10.1016/j.cell.2009.02.024 PMC2894612 19269363

[24] Ho Samantha SW Zhang Wendy YL Tan Nikki Yi J Khatoo M Suter Manuel A Tripathi S. The DNA Structure-Specific Endonuclease MUS81 Mediates DNA Sensor STING-Dependent Host Rejection of Prostate Cancer Cells. Immunity (2016) 44(5):1177–89. doi: 10.1016/j.immuni.2016.04.010 27178469

[25] Mackenzie KJ Carroll P Martin C-A Murina O Fluteau A Simpson DJ. cGAS Surveillance of Micronuclei Links Genome Instability to Innate Immunity. Nature (2017) 548(7668):461–5. doi: 10.1038/nature23449 PMC5870830 28738408

[26] Kitajima S Ivanova E Guo S Yoshida R Campisi M Sundararaman SK. Suppression of STING Associated With LKB1 Loss in KRAS-Driven Lung Cancer. Cancer Discov (2019) 9(1):34–45. doi: 10.1158/2159-8290.CD-18-0689 30297358 PMC6328329

[27] Riley JS Quarato G Cloix C Lopez J O'Prey J Pearson M. Mitochondrial Inner Membrane Permeabilisation Enables mtDNA Release During Apoptosis. EMBO J (2018) 37(17):e99238. doi: 10.15252/embj.201899238 30049712 PMC6120664

[28] Tan AS Baty JW Dong L-F Bezawork-Geleta A Endaya B Goodwin J. Mitochondrial Genome Acquisition Restores Respiratory Function and Tumorigenic Potential of Cancer Cells Without Mitochondrial DNA. Cell Metab (2015) 21(1):81–94. doi: 10.1016/j.cmet.2014.12.003 25565207

[29] Kwon J Bakhoum SF. The Cytosolic DNA-Sensing cGAS-STING Pathway in Cancer. Cancer Discov (2020) 10(1):26–39. doi: 10.1158/2159-8290.CD-19-0761 31852718 PMC7151642

[30] Sharma A Johnson A. Exosome DNA: Critical Regulator of Tumor Immunity and a Diagnostic Biomarker. J Cell Physiol (2020) 235(3):1921–32. doi: 10.1002/jcp.29153 31512231

[31] Schlacher K Wu H Jasin M. A Distinct Replication Fork Protection Pathway Connects Fanconi Anemia Tumor Suppressors to RAD51-Brca1/2. Cancer Cell (2012) 22(1):106–16. doi: 10.1016/j.ccr.2012.05.015 PMC3954744 22789542

[32] Parkes EE Walker SM Taggart LE McCabe N Knight LA Wilkinson R. Activation of STING-Dependent Innate Immune Signaling By S-Phase-Specific DNA Damage in Breast Cancer. J Natl Cancer Inst (2017) 109(1):djw199. doi: 10.1093/jnci/djw199 PMC5441301 27707838

[33] Koboldt DC Fulton RS McLellan MD Schmidt H Kalicki-Veize J McMichael JF. Comprehensive Molecular Portraits of Human Breast Tumours. Nature (2012) 490(7418):61–70. doi: 10.1038/nature11412 23000897 PMC3465532

[34] Wu S-Y Xiao Y Wei J-L Xu X-E Jin X Hu X. MYC Suppresses STING-Dependent Innate Immunity by Transcriptionally Upregulating DNMT1 in Triple-Negative Breast Cancer. J Immunother Cancer (2021) 9(7):e002528. doi: 10.1136/jitc-2021-002528 34321275 PMC8320259

[35] Meng F Yu Z Zhang D Chen S Guan H Zhou R. Induced Phase Separation of Mutant NF2 Imprisons the cGAS-STING Machinery to Abrogate Antitumor Immunity. Mol Cell (2021) 81(20):4147–64.e7. doi: 10.1016/j.molcel.2021.07.040 34453890

[36] Liu H Zhang H Wu X Ma D Wu J Wang L. Nuclear cGAS Suppresses DNA Repair and Promotes Tumorigenesis. Nature (2018) 563(7729):131–6. doi: 10.1038/s41586-018-0629-6 30356214

[37] Ranoa DRE Widau RC Mallon S Parekh AD Nicolae CM Huang X. STING Promotes Homeostasis *via* Regulation of Cell Proliferation and Chromosomal Stability. Cancer Res (2019) 79(7):1465–79. doi: 10.1158/0008-5472.CAN-18-1972 PMC6445702 30482772

[38] Leon KE Buj R Lesko E Dahl ES Chen C-W Tangudu NK. DOT1L Modulates the Senescence-Associated Secretory Phenotype Through Epigenetic Regulation of IL1A. J Cell Biol (2021) 220(8):e202008101. doi: 10.1083/jcb.202008101 34037658 PMC8160577

[39] Coppé J-P Desprez P-Y Krtolica A Campisi J. The Senescence-Associated Secretory Phenotype: The Dark Side of Tumor Suppression. Annu Rev Pathol (2010) 5:99–118. doi: 10.1146/annurev-pathol-121808-102144 20078217 PMC4166495

[40] Ivanov A Pawlikowski J Manoharan I van Tuyn J Nelson DM Rai TS. Lysosome-Mediated Processing of Chromatin in Senescence. J Cell Biol (2013) 202(1):129–43. doi: 10.1083/jcb.201212110 PMC3704985 23816621

[41] Glück S Guey B Gulen MF Wolter K Kang T-W Schmacke NA. Innate Immune Sensing of Cytosolic Chromatin Fragments Through cGAS Promotes Senescence. Nat Cell Biol (2017) 19(9):1061–70. doi: 10.1038/ncb3586 PMC5826565 28759028

[42] Yang B Dan X Hou Y Lee J-H Wechter N Krishnamurthy S. NAD(+) Supplementation Prevents STING-Induced Senescence in Ataxia Telangiectasia by Improving Mitophagy. Aging Cell (2021) 20(4):e13329–e. doi: 10.1111/acel.13329 PMC8045911 33734555

[43] Coppé J-P Patil CK Rodier F Sun Y Muñoz DP Goldstein J. Senescence-Associated Secretory Phenotypes Reveal Cell-Nonautonomous Functions of Oncogenic RAS and the P53 Tumor Suppressor. PLoS Biol (2008) 6(12):2853–68. doi: 10.1371/journal.pbio.0060301 PMC2592359 19053174

[44] Gulen MF Koch U Haag SM Schuler F Apetoh L Villunger A. Signalling Strength Determines Proapoptotic Functions of STING. Nat Commun (2017) 8(1):427. doi: 10.1038/s41467-017-00573-w 28874664 PMC5585373

[45] Zheng J Mo J Zhu T Zhuo W Yi Y Hu S. Comprehensive Elaboration of the cGAS-STING Signaling Axis in Cancer Development and Immunotherapy. Mol Cancer (2020) 19(1):133. doi: 10.1186/s12943-020-01250-1 32854711 PMC7450153

[46] Fuertes MB Kacha AK Kline J Woo S-R Kranz DM Murphy KM. Host Type I IFN Signals Are Required for Antitumor CD8+ T Cell Responses Through CD8{alpha}+ Dendritic Cells. J Exp Med (2011) 208(10):2005–16. doi: 10.1084/jem.20101159 PMC3182064 21930765

[47] Diamond JM Vanpouille-Box C Spada S Rudqvist N-P Chapman JR Ueberheide BM. Exosomes Shuttle TREX1-Sensitive IFN-Stimulatory dsDNA From Irradiated Cancer Cells to DCs. Cancer Immunol Res (2018) 6(8):910–20. doi: 10.1158/2326-6066.CIR-17-0581 PMC6072562 29907693

[48] Padovan E Spagnoli GC Ferrantini M Heberer M. IFN-Alpha2a Induces IP-10/CXCL10 and MIG/CXCL9 Production in Monocyte-Derived Dendritic Cells and Enhances Their Capacity to Attract and Stimulate CD8+ Effector T Cells. J Leukoc Biol (2002) 71(4):669–76. doi: 10.1189/jlb.71.4.669 11927654

[49] An X Zhu Y Zheng T Wang G Zhang M Li J. An Analysis of the Expression and Association With Immune Cell Infiltration of the cGAS/STING Pathway in Pan-Cancer. Mol Ther Nucleic Acids (2019) 14:80–9. doi: 10.1016/j.omtn.2018.11.003 PMC6305687 30583098

[50] Chen DS Mellman I. Oncology Meets Immunology: The Cancer-Immunity Cycle. Immunity (2013) 39(1):1–10. doi: 10.1016/j.immuni.2013.07.012 23890059

[51] Li W Lu L Lu J Wang X Yang C Jin J. cGAS-STING-Mediated DNA Sensing Maintains CD8 T Cell Stemness and Promotes Antitumor T Cell Therapy. Sci Transl Med (2020) 12(549):eaay9013. doi: 10.1126/scitranslmed.aay9013 32581136

[52] Ritchie C Cordova AF Hess GT Bassik MC Li L. SLC19A1 Is an Importer of the Immunotransmitter cGAMP. Mol Cell (2019) 75(2):372–81.e5. doi: 10.1016/j.molcel.2019.05.006 31126740 PMC6711396

[53] Lopez JA Jenkins MR Rudd-Schmidt JA Brennan AJ Danne JC Mannering SI. Rapid and Unidirectional Perforin Pore Delivery at the Cytotoxic Immune Synapse. J Immunol (2013) 191(5):2328–34. doi: 10.4049/jimmunol.1301205 23885110

[54] Deng L Liang H Xu M Yang X Burnette B Arina A. STING-Dependent Cytosolic DNA Sensing Promotes Radiation-Induced Type I Interferon-Dependent Antitumor Immunity in Immunogenic Tumors. Immunity (2014) 41(5):843–52. doi: 10.1016/j.immuni.2014.10.019 PMC5155593 25517616

[55] Ohkuri T Kosaka A Ishibashi K Kumai T Hirata Y Ohara K. Intratumoral Administration of cGAMP Transiently Accumulates Potent Macrophages for Anti-Tumor Immunity at a Mouse Tumor Site. Cancer Immunol Immunother (2017) 66(6):705–16. doi: 10.1007/s00262-017-1975-1 PMC11028681 28243692

[56] Veenhuis RT Freeman ZT Korleski J Cohen LK Massaccesi G Tomasi A. HIV-Antibody Complexes Enhance Production of Type I Interferon by Plasmacytoid Dendritic Cells. J Clin Invest (2017) 127(12):4352–64. doi: 10.1172/JCI95375 PMC5707144 29083319

[57] Demaria O De Gassart A Coso S Gestermann N Di Domizio J Flatz L. STING Activation of Tumor Endothelial Cells Initiates Spontaneous and Therapeutic Antitumor Immunity. Proc Natl Acad Sci USA (2015) 112(50):15408–13. doi: 10.1073/pnas.1512832112 PMC4687570 26607445

[58] Yang H Lee WS Kong SJ Kim CG Kim JH Chang SK. STING Activation Reprograms Tumor Vasculatures and Synergizes With VEGFR2 Blockade. J Clin Invest (2019) 129(10):4350–64. doi: 10.1172/JCI125413 PMC6763266 31343989

[59] Bakhoum SF Ngo B Laughney AM Cavallo J-A Murphy CJ Ly P. Chromosomal Instability Drives Metastasis Through a Cytosolic DNA Response. Nature (2018) 553(7689):467–72. doi: 10.1038/nature25432 PMC5785464 29342134

[60] Woo S-R Fuertes MB Corrales L Spranger S Furdyna MJ Leung MYK. STING-Dependent Cytosolic DNA Sensing Mediates Innate Immune Recognition of Immunogenic Tumors. Immunity (2014) 41(5):830–42. doi: 10.1016/j.immuni.2014.10.017 PMC4384884 25517615

[61] Decout A Katz JD Venkatraman S Ablasser A. The cGAS-STING Pathway as a Therapeutic Target in Inflammatory Diseases. Nat Rev Immunol (2021) 21(9):548–9. doi: 10.1038/s41577-021-00524-z PMC8029610 33833439

[62] Li J Duran MA Dhanota N Chatila WK Bettigole SE Kwon J. Metastasis and Immune Evasion From Extracellular cGAMP Hydrolysis. Cancer Discov (2021) 11(5):1212–27. doi: 10.1158/2159-8290.CD-20-0387 PMC8102348 33372007

[63] Ahn J Xia T Konno H Konno K Ruiz P Barber GN. Inflammation-Driven Carcinogenesis Is Mediated Through STING. Nat Commun (2014) 5:5166. doi: 10.1038/ncomms6166 25300616 PMC4998973

[64] Lemos H Mohamed E Huang L Ou R Pacholczyk G Arbab AS. STING Promotes the Growth of Tumors Characterized by Low Antigenicity *via* IDO Activation. Cancer Res (2016) 76(8):2076–81. doi: 10.1158/0008-5472.CAN-15-1456 PMC4873329 26964621

[65] Chen Q Boire A Jin X Valiente M Er EE Lopez-Soto A. Carcinoma-Astrocyte Gap Junctions Promote Brain Metastasis by cGAMP Transfer. Nature (2016) 533(7604):493–8. doi: 10.1038/nature18268 PMC5021195 27225120

[66] Fitzgerald KA McWhirter SM Faia KL Rowe DC Latz E Golenbock DT. IKKepsilon and TBK1 Are Essential Components of the IRF3 Signaling Pathway. Nat Immunol (2003) 4(5):491–6. doi: 10.1038/ni921 12692549

[67] Abe T Barber GN. Cytosolic-DNA-Mediated, STING-Dependent Proinflammatory Gene Induction Necessitates Canonical NF-κb Activation Through TBK1. J Virol (2014) 88(10):5328–41. doi: 10.1128/JVI.00037-14 PMC4019140 24600004

[68] Marcus A Mao AJ Lensink-Vasan M Wang L Vance RE Raulet DH. Tumor-Derived cGAMP Triggers a STING-Mediated Interferon Response in Non-Tumor Cells to Activate the NK Cell Response. Immunity (2018) 49(4):754–63.e4. doi: 10.1016/j.immuni.2018.09.016 30332631 PMC6488306

[69] Tas SW Vervoordeldonk MJ Hajji N Schuitemaker JHN van der Sluijs KF May MJ. Noncanonical NF-kappaB Signaling in Dendritic Cells Is Required for Indoleamine 2,3-Dioxygenase (IDO) Induction and Immune Regulation. Blood (2007) 110(5):1540–9. doi: 10.1182/blood-2006-11-056010 17483297

[70] Matsuura A Tsukada M Wada Y Ohsumi Y. Apg1p, a Novel Protein Kinase Required for the Autophagic Process in Saccharomyces Cerevisiae. Gene (1997) 192(2):245–50. doi: 10.1016/S0378-1119(97)00084-X 9224897

[71] Lazarus MB Novotny CJ Shokat KM. Structure of the Human Autophagy Initiating Kinase ULK1 in Complex With Potent Inhibitors. ACS Chem Biol (2015) 10(1):257–61. doi: 10.1021/cb500835z PMC4301081 25551253

[72] Gui X Yang H Li T Tan X Shi P Li M. Autophagy Induction *via* STING Trafficking Is a Primordial Function of the cGAS Pathway. Nature (2019) 567(7747):262–6. doi: 10.1038/s41586-019-1006-9 PMC9417302 30842662

[73] Nassour J Radford R Correia A Fusté JM Schoell B Jauch A. Autophagic Cell Death Restricts Chromosomal Instability During Replicative Crisis. Nature (2019) 565(7741):659–63. doi: 10.1038/s41586-019-0885-0 PMC6557118 30675059

[74] Song M Sandoval TA Chae C-S Chopra S Tan C Rutkowski MR. Ire1α-XBP1 Controls T Cell Function in Ovarian Cancer by Regulating Mitochondrial Activity. Nature (2018) 562(7727):423–8. doi: 10.1038/s41586-018-0597-x PMC6237282 30305738

[75] Wang Y Luo J Alu A Han X Wei Y Wei X. cGAS-STING Pathway in Cancer Biotherapy. Mol Cancer (2020) 19(1):136. doi: 10.1186/s12943-020-01247-w 32887628 PMC7472700

[76] Ross P Weinhouse H Aloni Y Michaeli D Weinberger-Ohana P Mayer R. Regulation of Cellulose Synthesis in Acetobacter Xylinum by Cyclic Diguanylic Acid. Nature (1987) 325(6101):279–81. doi: 10.1038/325279a0 18990795

[77] Ohkuri T Ghosh A Kosaka A Zhu J Ikeura M David M. STING Contributes to Antiglioma Immunity *via* Triggering Type I IFN Signals in the Tumor Microenvironment. Cancer Immunol Res (2014) 2(12):1199–208. doi: 10.1158/2326-6066.CIR-14-0099 PMC4258479 25300859

[78] Chandra D Quispe-Tintaya W Jahangir A Asafu-Adjei D Ramos I Sintim HO. STING Ligand C-Di-GMP Improves Cancer Vaccination Against Metastatic Breast Cancer. Cancer Immunol Res (2014) 2(9):901–10. doi: 10.1158/2326-6066.CIR-13-0123 PMC4264585 24913717

[79] Li T Cheng H Yuan H Xu Q Shu C Zhang Y. Antitumor Activity of cGAMP *via* Stimulation of cGAS-cGAMP-STING-IRF3 Mediated Innate Immune Response. Sci Rep (2016) 6:19049. doi: 10.1038/srep19049 26754564 PMC4709567

[80] Dorostkar F Arashkia A Roohvand F Shoja Z Navari M Mashhadi Abolghasem Shirazi M. Co-Administration of 2'3'-cGAMP STING Activator and CpG-C Adjuvants With a Mutated Form of HPV 16 E7 Protein Leads to Tumor Growth Inhibition in the Mouse Model. Infect Agent Cancer (2021) 16(1):7. doi: 10.1186/s13027-021-00346-7 33499895 PMC7836183

[81] Tang C-HA Zundell JA Ranatunga S Lin C Nefedova Y Del Valle JR. Agonist-Mediated Activation of STING Induces Apoptosis in Malignant B Cells. Cancer Res (2016) 76(8):2137–52. doi: 10.1158/0008-5472.CAN-15-1885 PMC4873432 26951929

[82] Ablasser A Goldeck M Cavlar T Deimling T Witte G Röhl I. cGAS Produces a 2'-5'-Linked Cyclic Dinucleotide Second Messenger That Activates STING. Nature (2013) 498(7454):380–4. doi: 10.1038/nature12306 PMC4143541 23722158

[83] Corrales L Glickman LH McWhirter SM Kanne DB Sivick KE Katibah GE. Direct Activation of STING in the Tumor Microenvironment Leads to Potent and Systemic Tumor Regression and Immunity. Cell Rep (2015) 11(7):1018–30. doi: 10.1016/j.celrep.2015.04.031 PMC4440852 25959818

[84] Lee SJ Yang H Kim WR Lee YS Lee WS Kong SJ. STING Activation Normalizes the Intraperitoneal Vascular-Immune Microenvironment and Suppresses Peritoneal Carcinomatosis of Colon Cancer. J Immunother Cancer (2021) 9(6):e002195. doi: 10.1136/jitc-2020-002195 34145029 PMC8215239

[85] Berry S Giraldo N Nguyen P Green B Xu H Ogurtsova A. Correction to: 33rd Annual Meeting & Pre-Conference Programs of the Society for Immunotherapy of Cancer (SITC 2018). J Immunother Cancer (2019) 7(1):46. doi: 10.1186/s40425-019-0519-y 30760319 PMC6373015

[86] Ager CR Zhang H Wei Z Jones P Curran MA Di Francesco ME. Discovery of IACS-8803 and IACS-8779, Potent Agonists of Stimulator of Interferon Genes (STING) With Robust Systemic Antitumor Efficacy. Bioorg Med Chem Lett (2019) 29(20):126640. doi: 10.1016/j.bmcl.2019.126640 31500996 PMC6993876

[87] Harrington KJ Brody J Ingham M Strauss J Cemerski S Wang M. LBA15 - Preliminary Results of the First-in-Human (FIH) Study of MK-1454, an Agonist of Stimulator of Interferon Genes (STING), as Monotherapy or in Combination With Pembrolizumab (Pembro) in Patients With Advanced Solid Tumors or Lymphomas. Ann Oncol (2018) 29:viii712. doi: 10.1093/annonc/mdy424.015

[88] Daei Farshchi Adli A Jahanban-Esfahlan R Seidi K Samandari-Rad S Zarghami N. An Overview on Vadimezan (DMXAA): The Vascular Disrupting Agent. Chem Biol Drug Des (2018) 91(5):996–1006. doi: 10.1111/cbdd.13166 29288534

[89] Pham MH Dauzonne D Chabot GG. Not Flavone-8-Acetic Acid (FAA) But its Murine Metabolite 6-OH-FAA Exhibits Remarkable Antivascular Activities. Vitro Anticancer Drugs (2016) 27(5):398–406. doi: 10.1097/CAD.0000000000000341 26901071

[90] Zaharko DS Grieshaber CK Plowman J Cradock JC. Therapeutic and Pharmacokinetic Relationships of Flavone Acetic Acid: An Agent With Activity Against Solid Tumors. Cancer Treat Rep (1986) 70(12):1415–21. doi: 10.1016/0304-3835(86)90075-3 3791254

[91] Kerr DJ Kaye SB. Flavone Acetic Acid–Preclinical and Clinical Activity. Eur J Cancer Clin Oncol (1989) 25(9):1271–2. doi: 10.1016/0277-5379(89)90072-2 2680512

[92] Ramanjulu JM Pesiridis GS Yang J Concha N Singhaus R Zhang S-Y. Design of Amidobenzimidazole STING Receptor Agonists With Systemic Activity. Nature (2018) 564(7736):439–43. doi: 10.1038/s41586-018-0705-y 30405246

[93] Lomax ME Folkes LK O'Neill P. Biological Consequences of Radiation-Induced DNA Damage: Relevance to Radiotherapy. Clin Oncol (R Coll Radiol) (2013) 25(10):578–85. doi: 10.1016/j.clon.2013.06.007 23849504

[94] Chen J Markelc B Kaeppler J Ogundipe VML Cao Y McKenna WG. STING-Dependent Interferon-λ1 Induction in HT29 Cells, a Human Colorectal Cancer Cell Line, After Gamma-Radiation. Int J Radiat Oncol Biol Phys (2018) 101(1):97–106. doi: 10.1016/j.ijrobp.2018.01.091 29619982

[95] Barcellos-Hoff MH Derynck R Tsang ML Weatherbee JA. Transforming Growth Factor-Beta Activation in Irradiated Murine Mammary Gland. J Clin Invest (1994) 93(2):892–9. doi: 10.1172/JCI117045 PMC293960 8113421

[96] Schaue D Comin-Anduix B Ribas A Zhang L Goodglick L Sayre JW. T-Cell Responses to Survivin in Cancer Patients Undergoing Radiation Therapy. Clin Cancer Res an Off J Am Assoc Cancer Res (2008) 14(15):4883–90. doi: 10.1158/1078-0432.CCR-07-4462 PMC2748652 18676762

[97] Chiang C-S Fu SY Wang S-C Yu C-F Chen F-H Lin C-M. Irradiation Promotes an M2 Macrophage Phenotype in Tumor Hypoxia. Front Oncol (2012) 2:89. doi: 10.3389/fonc.2012.00089 22888475 PMC3412458

[98] Vanpouille-Box C Alard A Aryankalayil MJ Sarfraz Y Diamond JM Schneider RJ. DNA Exonuclease Trex1 Regulates Radiotherapy-Induced Tumour Immunogenicity. Nat Commun (2017) 8:15618. doi: 10.1038/ncomms15618 28598415 PMC5472757

[99] Grabosch S Bulatovic M Zeng F Ma T Zhang L Ross M. Cisplatin-Induced Immune Modulation in Ovarian Cancer Mouse Models With Distinct Inflammation Profiles. Oncogene (2019) 38(13):2380–93. doi: 10.1038/s41388-018-0581-9 PMC6440870 30518877

[100] Luthra P Aguirre S Yen BC Pietzsch CA Sanchez-Aparicio MT Tigabu B. Topoisomerase II Inhibitors Induce DNA Damage-Dependent Interferon Responses Circumventing Ebola Virus Immune Evasion. mBio (2017) 8(2):e00368–17. doi: 10.1128/mBio.00368-17 PMC5380843 28377530

[101] Liu Y Crowe WN Wang L Lu Y Petty WJ Habib AA. An Inhalable Nanoparticulate STING Agonist Synergizes With Radiotherapy to Confer Long-Term Control of Lung Metastases. Nat Commun (2019) 10(1):5108. doi: 10.1038/s41467-019-13094-5 31704921 PMC6841721

[102] Peggs KS Quezada SA Chambers CA Korman AJ Allison JP. Blockade of CTLA-4 on Both Effector and Regulatory T Cell Compartments Contributes to the Antitumor Activity of Anti-CTLA-4 Antibodies. J Exp Med (2009) 206(8):1717–25. doi: 10.1084/jem.20082492 PMC2722174 19581407

[103] Francisco LM Salinas VH Brown KE Vanguri VK Freeman GJ Kuchroo VK. PD-L1 Regulates the Development, Maintenance, and Function of Induced Regulatory T Cells. J Exp Med (2009) 206(13):3015–29. doi: 10.1084/jem.20090847 PMC2806460 20008522

[104] Pardoll DM. The Blockade of Immune Checkpoints in Cancer Immunotherapy. Nat Rev Cancer (2012) 12(4):252–64. doi: 10.1038/nrc3239 PMC4856023 22437870

[105] Motedayen Aval L Pease JE Sharma R Pinato DJ. Challenges and Opportunities in the Clinical Development of STING Agonists for Cancer Immunotherapy. J Clin Med (2020) 9(10):3323. doi: 10.3390/jcm9103323 PMC7602874 33081170

[106] Dorta-Estremera S Hegde VL Slay RB Sun R Yanamandra AV Nicholas C. Targeting Interferon Signaling and CTLA-4 Enhance the Therapeutic Efficacy of Anti-PD-1 Immunotherapy in Preclinical Model of HPV Oral Cancer. J Immunother Cancer (2019) 7(1):252. doi: 10.1186/s40425-019-0728-4 31533840 PMC6749627

[107] Li K Ye Y Liu L Sha Q Wang X Jiao T. The Lipid Platform Increases the Activity of STING Agonists to Synergize Checkpoint Blockade Therapy Against Melanoma. Biomater Sci (2021) 9(3):765–73. doi: 10.1039/d0bm00870b 33201161

[108] Shae D Baljon JJ Wehbe M Christov PP Becker KW Kumar A. Co-Delivery of Peptide Neoantigens and Stimulator of Interferon Genes Agonists Enhances Response to Cancer Vaccines. ACS Nano (2020) 14(8):9904–16. doi: 10.1021/acsnano.0c02765 PMC7775800 32701257

[109] Fu J Kanne DB Leong M Glickman LH McWhirter SM Lemmens E. STING Agonist Formulated Cancer Vaccines Can Cure Established Tumors Resistant to PD-1 Blockade. Sci Transl Med (2015) 7(283):283ra52. doi: 10.1126/scitranslmed.aaa4306 PMC4504692 25877890

[110] Hombach A Wieczarkowiecz A Marquardt T Heuser C Usai L Pohl C. Tumor-Specific T Cell Activation by Recombinant Immunoreceptors: CD3 Zeta Signaling and CD28 Costimulation Are Simultaneously Required for Efficient IL-2 Secretion and Can be Integrated Into One Combined CD28/CD3 Zeta Signaling Receptor Molecule. J Immunol (2001) 167(11):6123–31. doi: 10.4049/jimmunol.167.11.6123 11714771

[111] Patel SD Moskalenko M Smith D Maske B Finer MH McArthur JG. Impact of Chimeric Immune Receptor Extracellular Protein Domains on T Cell Function. Gene Ther (1999) 6(3):412–9. doi: 10.1038/sj.gt.3300831 10435091

[112] Ma S Li X Wang X Cheng L Li Z Zhang C. Current Progress in CAR-T Cell Therapy for Solid Tumors. Int J Biol Sci (2019) 15(12):2548–60. doi: 10.7150/ijbs.34213 PMC6854376 31754328

[113] Xu N Palmer DC Robeson AC Shou P Bommiasamy H Laurie SJ. STING Agonist Promotes CAR T Cell Trafficking and Persistence in Breast Cancer. J Exp Med (2021) 218(2):e20200844. doi: 10.1084/jem.20200844 33382402 PMC7780733

[114] Xu W Atkinson VG Menzies AM. Intratumoural Immunotherapies in Oncology. Eur J Cancer (2020) 127:1–11. doi: 10.1016/j.ejca.2019.12.007 31962197

[115] Broman KK Zager JS. An Evaluation of Talimogene Laherparepvec for the Treatment of Melanoma. Expert Opin Biol Ther (2020) 20(1):9–14. doi: 10.1080/14712598.2020.1689951 31690129

[116] Kaufman HL Kim DW DeRaffele G Mitcham J Coffin RS Kim-Schulze S. Local and Distant Immunity Induced by Intralesional Vaccination With an Oncolytic Herpes Virus Encoding GM-CSF in Patients With Stage IIIc and IV Melanoma. Ann Surg Oncol (2010) 17(3):718–30. doi: 10.1245/s10434-009-0809-6 19915919

[117] Xia T Konno H Barber GN. Recurrent Loss of STING Signaling in Melanoma Correlates With Susceptibility to Viral Oncolysis. Cancer Res (2016) 76(22):6747–59. doi: 10.1158/0008-5472.CAN-16-1404 27680683

[118] Wu J-J Zhao L Hu H-G Li W-H Li Y-M. Agonists and Inhibitors of the STING Pathway: Potential Agents for Immunotherapy. Med Res Rev (2020) 40(3):1117–41. doi: 10.1002/med.21649 31793026

[119] Siu T Altman MD Baltus GA Childers M Ellis JM Gunaydin H. Discovery of a Novel cGAMP Competitive Ligand of the Inactive Form of STING. ACS Med Chem Lett (2019) 10(1):92–7. doi: 10.1021/acsmedchemlett.8b00466 PMC6331172 30655953

[120] Li S Hong Z Wang Z Li F Mei J Huang L. The Cyclopeptide Astin C Specifically Inhibits the Innate Immune CDN Sensor STING. Cell Rep (2018) 25(12):3405–21.e7. doi: 10.1016/j.celrep.2018.11.097 30566866

[121] Haag SM Gulen MF Reymond L Gibelin A Abrami L Decout A. Targeting STING With Covalent Small-Molecule Inhibitors. Nature (2018) 559(7713):269–73. doi: 10.1038/s41586-018-0287-8 29973723

[122] Hansen AL Buchan GJ Rühl M Mukai K Salvatore SR Ogawa E. Nitro-Fatty Acids Are Formed in Response to Virus Infection and Are Potent Inhibitors of STING Palmitoylation and Signaling. Proc Natl Acad Sci USA (2018) 115(33):E7768–E75. doi: 10.1073/pnas.1806239115 PMC6099880 30061387

